# Congenital anomalies in pregnancies with overt and pregestational type 2 diabetes: a gray portrayal from a cohort in Brazil

**DOI:** 10.1186/s13098-024-01376-y

**Published:** 2024-07-11

**Authors:** Maria Amélia A Campos, Maria Lúcia R Oppermann, Maria Teresa V Sanseverino, Giulia L Guerra, Vânia N Hirakata, Angela J Reichelt

**Affiliations:** 1 Serviço de Endocrinologia, Hospital Nossa Senhora da Conceição, Porto Alegre, Brazil; 2 Serviço de Ginecologia e Obstetrícia, Hospital de Clínicas de Porto Alegre, and Faculdade de Medicina, Universidade Federal do Rio Grande do Sul, Porto Alegre, Brazil; 3 Serviço de Genética Médica, Hospital de Clínicas de Porto Alegre, and Faculdade de Medicina da Pontifícia Universidade Católica, Porto Alegre, Brazil; 4 Faculdade de Medicina, Universidade Feevale, Novo Hamburgo, Brazil; 5 Unidade de Bioestatística e Análise de Dados, Hospital de Clínicas de Porto Alegre, and Programa de Pós-graduação em Cardiologia e Ciências Cardiovasculares, Universidade Federal do Rio Grande do Sul, Porto Alegre, Brazil; 6 Serviço de Endocrinologia e Metabologia, Hospital de Clínicas de Porto Alegre, and Programa de Pós‑Graduação em Ciências Médicas: Endocrinologia, Universidade Federal do Rio Grande do Sul Porto Alegre, Porto Alegre, Rio Grande do Sul Brazil

**Keywords:** Congenital anomaly, Type 2 diabetes during pregnancy

## Abstract

**Objective:**

To describe the frequency and types of congenital anomalies and associated risk factors in Brazilian women with type 2 diabetes.

**Methods:**

In this retrospective cohort study between 2005 and 2021, we included all pregnant participants with type 2 diabetes from the two major public hospitals in southern Brazil. We collected data from the electronic hospital records. Congenital anomalies were classified by the 10^th^ revised International Classification of Diseases, Q chapter, enhanced by the EUROCAT registry classification, and categorized by type and gravity. We used multiple Poisson regression with robust estimates to estimate risks.

**Results:**

Among 648 participants, we excluded 19, and 62 were lost to follow-up; therefore, we included 567 participants. Overt diabetes arose in 191 participants (33.7%, 95% CI 30.0% – 38.0%). Less than 20% of the participants supplemented folate. Congenital anomalies occurred in 78 neonates (13.8%, CI 11.0 − 16.9%), 73 babies (93.6%) presented major anomalies, and 20 (10.5%) cases occurred in participants with overt diabetes. Cardiac anomalies were the most frequent (43 isolated and 12 combined). Pre-eclampsia was associated with an increased risk in the analyses including all women (adjusted RR 1.87 (95% CI 1.23–2.85), *p* = 0.003), but not in analyses including only women with an HbA1c measured up to the 14^th^ gestational age. HbA1c, either measured at any time in pregnancy (adjusted RR 1.21 (95% CI 1.10–1.33), *p* < 0.001) or up to the first 14 weeks (adjusted RR 1.22, 95% CI 1.10–1.35, *p* < 0.001) was the only sustained risk factor. Risk factors such as maternal age, obesity, diabetes diagnosis, or use of antidiabetic medications were not associated with congenital anomalies.

**Conclusion:**

We found a high frequency of congenital anomalies associated with poor maternal glycemic control and revealed an almost universal lack of preconception care. An urgent call to action is mandatory for the reversal of this gray scenario.

**Supplementary Information:**

The online version contains supplementary material available at 10.1186/s13098-024-01376-y.

## Background

The sharp increase in the worldwide prevalence of obesity seen in recent decades, with the accompanying rise in type 2 diabetes diagnosis, has led to a shift toward the diagnosis of hyperglycemia at younger ages, affecting women at childbearing ages [1]. In Brazil, rates of obesity are increasing - up to 27.5% in women aged 25 to 44 years - whereas rates of diabetes increase from 0.6 to 6.3% in women aged 18 to 44 years - with an increase paralleling the age stratum [2]. The frequency of undiagnosed diabetes is high in our country, reaching 31.9% in adults aged 20 to 79 years [1]. We had previously reported a high frequency of overt diabetes, that is, the diagnosis of diabetes for the first time in pregnancy, among women with type 2 diabetes receiving prenatal care at high-risk facilities in our city: ~30% [3].

Hyperglycemia is a recognized link in the genesis of congenital anomalies [4], the “structural or functional anomalies that occur during intrauterine life” [5], which can be identified during pregnancy or at any time after birth. Genetic and environmental factors are involved in the development of congenital anomalies. In addition to hyperglycemia, obesity, drugs, and environmental pollutants have also been implicated [6]. The mechanisms associated with hyperglycemia and congenital anomalies are not completely understood [7].

It is well-known that receiving preconception care favorably affects pregnancy outcomes in women with diabetes: the risk of birth defects decreased by 71% in women who prepared for pregnancy compared to those without preconception care [8]. Nevertheless, low rates of pregnancy preparation were consistently reported: only ~ 39% of women prepared for pregnancy in the cohort studies included in a meta-analysis [8].

There are scarce reports on the outcomes of pregnancies in women with type 2 diabetes in the country, generally including few participants during short periods of observation. In this context, we aimed to describe the frequency and categories of congenital anomalies and analyze the associated risk factors in a cohort of Brazilian women with type 2 diabetes.

## Methods

In this retrospective cohort, we enrolled all women with type 2 diabetes receiving prenatal care at the two major public hospitals in Porto Alegre, Brazil, from May 2005 to May 2021. Both hospitals deliver care to women with high-risk pregnancies, referred from the primary care setting of the national public health service network (Sistema Único de Saúde, SUS). Hospital de Clínicas de Porto Alegre (HCPA) is a university hospital where 2,871 deliveries took place in 2023 [9]; Hospital Nossa Senhora da Conceição is a tertiary school hospital in which more than 6,000 deliveries took place in 2022 [10].

The ethics committees of both hospitals approved the study protocol on July 28, 2016, and we registered the project in *Plataforma Brasil*, CAAE 57365016.3.0000.5327. All authors signed a data use agreement form to ensure the privacy of the data collected from medical registries.

For sample size calculation, we used the Power and Sample Size for Health Researchers (PSS_Health), available at https://hcpa-unidade-bioestatistica.shinyapps.io/PSS_Health/. Based on an expected prevalence of congenital anomalies of 6% [5], a 95% confidence interval, and a 2.5% margin of error, the determined sample size was 386 participants, at least.

Participants were eligible if they presented typical clinical features and a preconception diagnosis of type 2 diabetes or fulfilled the World Health Organization criteria for overt diabetes (fasting plasma glucose ≥ 126 mg/dl or 2-h plasma glucose after a 75 g load ≥ 200 mg/dl) [11] and/or the American Diabetes Association recommendation of glycated hemoglobin (Hba1c) ≥ 6.5% [12]. We excluded participants who had twin pregnancies, type 1 diabetes, clinical or laboratory features of latent autoimmune diabetes of adulthood, gestational diabetes, an uncertain diagnosis of hyperglycemia, or any chromosomal anomaly in the current pregnancy.

Multidisciplinary teams provided antenatal care to women with high-risk pregnancies at both hospitals. Previous publications with a focus on other objectives included part or the whole sample presented here [3, 13].

After approval of the study protocol, we retrieved data from the electronic medical records and transcribed them to a SPSS spreadsheet with structured variables. We collected self-reported information on diabetes diagnosis (pregestational or overt) and the informed pre-pregnancy weight. Chronic diabetes complications (albuminuria, retinopathy, neuropathy and macrovascular disease), use of tobacco or other illicit or non-illicit drugs, use of medication at conception (folate, oral antidiabetic agents and anti-hypertensive medications), personal history of hypertension or previous gestational diabetes, and delivery of a macrosomic baby (birth weight ≥ 4000 g) were treated as positive when available. We measured height at the first prenatal appointment, and weight at each visit and admission for delivery. Pregestational body mass index (BMI), calculated as the informed pregestational weight (kilograms) divided by the squared height (meters), was classified into normal BMI, overweight, or obesity [14]. Preeclampsia was defined as “gestational hypertension (systolic BP ≥ 140 mm Hg or diastolic BP ≥ 90 mm Hg at ≥ 20 wk 0 days of gestation)” plus clinic proteinuria or another maternal organ dysfunction [15]. The categories of birthweight were adequate for gestational age (AGA), small for gestational age (SGA), or large for gestational age (LGA) [16]. The neonatal medical team attending birth determined admission to the intensive care unit. We retrieved information on the congenital anomalies from the electronic records. The geneticist of the group (MTS) revised the classification of the anomalies according to the 10th revised International Classification of Diseases, Q chapter, enhanced by the classification adopted by the EUROCAT registry [17]. Congenital anomalies were categorized by type and severity. Major anomalies were those with “significant medical, surgical, social or cosmetic consequences for the affected individual, and typically require medical intervention” and minor, those “structural changes that pose little or no significant health problem and tend to have limited social or cosmetic consequences for the affected individual” [17, 18].

HbA1c was measured at booking, irrespective of gestational age, and was measured at least once after the 28th gestational week. Assays were conducted with high-performance liquid chromatography (Variant II Turbo HbA1c; BioRad, Hercules, CA, USA) in line with the National Glycohemoglobin Standardization Program guidelines (http://www.ngsp.org/ index. asp).

For analyses, we considered all missing information on medications, smoking status, alcohol use, and illicit drug use to be negative and acknowledged that reporting bias could have occurred. We classified women with underweight as the normal BMI category, as we had only four cases. The analyses with the use of metformin (no/yes) and sulfonylurea (no/yes) were performed exclusively on the group with known pregestational diabetes. We estimated the frequency of congenital anomalies with the respective 95% confidence intervals; and we compared several characteristics of the groups with and without anomalies by bivariate analysis using the Student’s t-test or the chi-square test (coupled with the Z test for comparison of proportions, with Bonferroni correction) as appropriate.

We evaluated risk factors associated with the presence (or not, reference category) of congenital anomalies (dependent variable) by multiple Poisson regression with robust estimates. In multiple Poisson regression risks are estimated for all the variables included in the models and the results, therefore, are fully adjusted for these variables. We included in the multivariable models those variables with a *p* < 0.2 in the bivariate analysis. We dichotomized variables as obesity (yes (present)/no (absent)), the timing of diabetes diagnosis (overt or pregestational diabetes); and for women with pregestational diabetes, diabetes diagnosis < 6 years or ≥ 6 years; all maternal and neonatal outcomes were dichotomized as yes (present)/no (absent).

Statistical analyses were performed with SPSS version 29.0 (SPSS, Chicago, IL, USA).

The data are expressed as the mean ± standard deviation (SD) or number (percentage).

Unless otherwise noted, the total number of cases is in the caption of each Table; when a variable presented missing values, we inserted a line below the results showing the number of cases effectively evaluated for that variable. The results of the multivariable analyses are expressed as the adjusted relative risk (aRR) and 95% confidence interval (CI).

The manuscript was written following the Strengthening the Reporting of Observational Studies in Epidemiology (STROBE) guidelines [19].

## Results

**Fig. 1:**
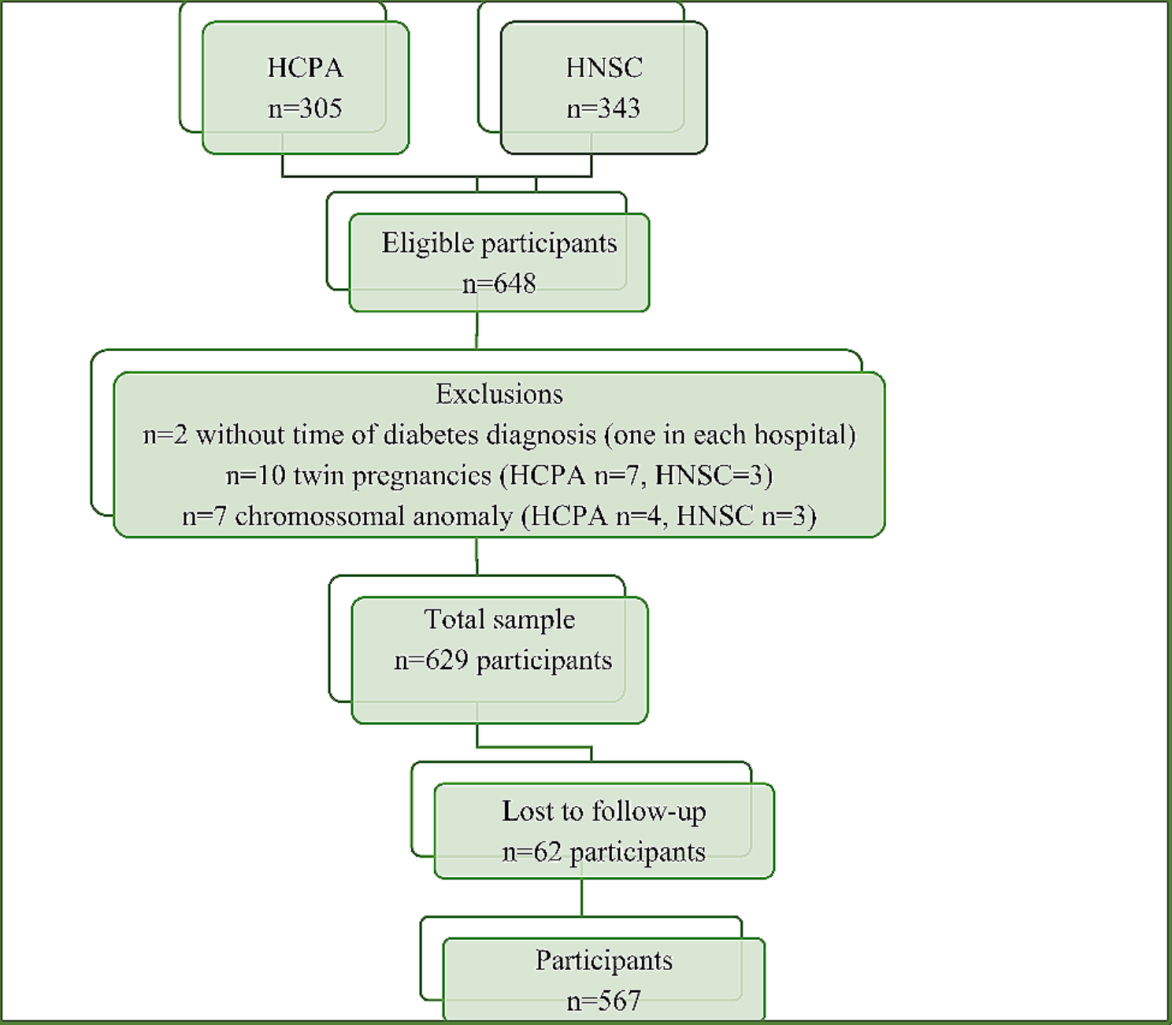
Flowchart of the study. HCPA: Hospital de Clínicas de Porto Alegre; HNSC: Hospital Nossa Senhora da Conceição

We enrolled 648 participants: 19 were excluded and 62 were lost to follow-up, as shown in the study flowchart (Fig. 1), leaving 567 participants. The main maternal baseline characteristics and perinatal outcomes are shown in Table 1. Overt diabetes was diagnosed in 191 women (33.7%, 95% CI 30% – 38%); median diabetes duration in those with known diabetes diagnosis was 4.0 years (2.0–7.0 years). We did not find differences in baseline characteristics between women who delivered babies with and without congenital anomalies, including smoking, alcohol use (data not shown because only 3 women referred regular use of alcohol), folic acid intake, or number of pregnancies (mean 3.0 ± 1.7 pregnancies) (Table 1). Adequate glycemic control in the first 14 gestational weeks was similar between groups, but more babies were affected in pregnancies with longer diabetes diagnosis.

**Table 1:** Characteristics of pregnancies in women with type 2 diabetes by congenital anomalies Porto Alegre, Brazil, 2005–2021

Characteristic		Congenital anomaly		
	All *n* = 567 (100.0)	Yes *n* = 78 (13.8)	No *n* = 489 (86.2)	p
**Baseline characteristics**				
Center				0.464
HCPA	258 (45.5)	32 (12.4)	226 (87.6)	
HNSC	309 (54.5)	46 (14.9)	263 (85.1)	
Age (years)	32.8 ± 5.9	32.4 ± 6.5	32.9 ± 5.8	0.567
Timing of diabetes diagnosis				0.136
overt	191 (33.7)	20 (10.5)	171 (89.5)	
pregestational	376 (66.3)	58 (15.4)	318 (84.6)	
	567	78	489	
Skin color				> 0.999
white	391 (69.0)	54 (13.8)	337 (86.2)	
non-white	176 (31.0)	24 (13.6)	152 (86.4)	
Schooling				0.777
≤ 11 years	538 (94.9)	73 (13.6)	465 (86.4)	
> 11 years	29 (5.1)	5 (17.2)	24 (82.8)	
Smoking				0.871
yes	50 (8.8)	6 (12.0)	44 (88.0)	
no	517 (91.2)	72 (13.9)	445 (86.1)	
Pre-pregnancy treatment				0.423
none	228 (40.6)	26 (11.4)	202 (88.6)	
diet only	21 (3.7)	1 (4.8)	20 (95.2)	
oral medication	240 (42.7)	39 (16.3)	201 (83.7)	
insulin	27 (4.8)	4 (14.8)	23 (85.2)	
oral + insulin	46 (8.2)	7 (15.2)	39 (84.8)	
	562	77	485	
Folic acid intake				0.551
yes	100 (17.6)	17 (17.0)	83 (83.0)	
no	84 (14.8)	10 (11.9)	74 (88.1)	
no information	383 (67.6)	51 (13.3)	332 (86.7)	
Anti-hypertensive drugs	100 (17.6)	12 (12.0)	88 (88.0)	0.688
	467 (82.4)	66 (14.1)	401 (85.9)	
Statins				> 0.999
yes	16 (2.8)	2 (12.5)	14 (87.5)	
no	551 (97.2)	76 (13.8)	475 (86.2)	
Diabetes complications				0.439
yes	36 (6.3)	7 (19.4)	29 (80.6)	
no	531 (93.7)	71 (13.4)	460 (86.6)	
Chronic hypertension				0.722
yes	147 (25.9)	22 (15.0)	125 (85.0)	
no	420 (74.1)	56 (13.3)	364 (86.7)	
BMI				
pregestational (kg/m^2^)	34.4 ± 7.8	33.8 ± 7.6	34.4 ± 7.9	0.524
categories				0.461
no obesity	162 (29.5)	26 (16.0)	136 (84.0)	
obesity	386 (70.5)	51 (13.2)	335 (86.8)	
	548	77	471	
Gestational age at booking (weeks)	20.4 ± 8.2	20.7 ± 8.1	20.4 ± 8.3	0.735
First HbA1c	7.3 ± 1.6	8.0 ± 2.1	7.2 ± 1.4	0.001
	563	78	485	
HbA1c before 14 weeks				0.656
< 6.5%	57 (23.8)	7 (12.3)	50 (87.7)	
≥ 6.5%	183 (76.2)	29 (15.8)	154 (84.2)	
	240	36	204	
**Pregnancy outcomes**				
Preeclampsia				0.004
yes	187 (33.4)	36 (19.3)	151 (80.7)	
no	373 (66.6)	38 (10.2)	335 (89.8)	
	560	74	486	
Pregnancy outcome				< 0.001
miscarriage	6 (1.1)	2 (33.3)^a b^	4 (66.7)^a b^	
liveborn	547 (96.4)	69 (12.6)^b^	478 (87.4)^b^	
stillborn	12 (2.1)	5 (41.7)^a^	7 (58.3)^a^	
delivery in another hospital	2 (0.4)	2 (100)^a^	0 (0)^a^	
Birthweight adequacy				0.572
SGA	28 (5.0)	5 (17.9)	23 (82.1)	
AGA	312 (56.3)	37 (11.9)	275 (88.1)	
LGA	215 (38.7)	30 (14.0)	185 (86.0)	
	555	72	483	
NICU admission				< 0.001
yes	230 (42.0)	49 (21.3)	181 (78.7)	
no	317 (58.0)	20 (6.3)	297 (93.7)	
	547	69	478	
Neonatal death				< 0.001
yes	12 (2.2)	8 (66.7)	4 (33.3)	
no	535 (97.8)	61 (11.4)	474 (88.6)	
	547	69	478	

HCPA: Hospital de Clínicas de Porto Alegre; HNSC: Hospital Nossa Senhora da Conceição; HbA1c: glycated hemoglobin; BMI: body mass index; SGA: small for gestational age; AGA: adequate for gestational age; LGA: large for gestational age; NICU: neonatal intensive care unit

The data are presented as the mean (standard deviation) or n (%)

The total number of cases is in the caption of the Table. We added a line with the actual number analyzed under the results of the variables with missing data

p values were calculated with the χ^2^ test for categorical variables and the Student’s t-test for continuous variables

^a b^ Different letters in the categories of the variable indicate statistical differences in the column, analyzed by the Z-test for proportion comparisons, corrected by Bonferroni (*p* < 0.05)

Congenital anomalies were diagnosed in 78 (13.8%, 95% CI 11.0% – 16.9%) neonates: in 73 babies (93.6%), anomalies were classified as major ones; 20 (10.5%) cases occurred in participants with overt diabetes and 58 (15.4%) occurred in those with pregestational type 2 diabetes (*p* = 0.136). The most frequent anomalies were cardiac (43 as isolated heart defects and 12 in combination with other organ defects), followed by neurologic (*n* = 10), renal (*n* = 7), and miscellaneous (*n* = 6) anomalies. The types of congenital anomalies according to timing of diabetes diagnosis and the classification as major or minor are shown in Suppl Table  1. Cardiac and neurologic anomalies were more common in babies of women with diabetes diagnosed before pregnancy (Suppl Table 1), in line with a higher mean HbA1c before 14 gestational weeks in pregnancies with major congenital anomalies than in those without anomalies (Suppl Table 1). The frequencies of congenital malformations were stable across time, irrespective of diabetes classification (Suppl Table 2 and Suppl Fig. 1).

Less than 20% of women used folic acid in the first trimester of pregnancy, and those who did began use after the diagnosis of pregnancy. Almost 20% of women used anti-hypertensive medication; few used statins. Among those with known diabetes before pregnancy, metformin was the oral antidiabetic more frequently used, ~ 70% in the groups with or without congenital anomalies, whereas 10% more women used sulfonylureas in the group delivering babies with congenital anomalies. In Suppl Tables 3 and 4, we present the univariable analysis for pregestational diabetes and HbA1c measured up to the 14^th^ gestational week, to further explore statistical differences regarding the main outcome. Obesity, although not significant, was included in the multivariable models, as it is a recognized risk factor for congenital anomalies.

The results of the multivariable analyses for any congenital anomaly (major or minor) risk are in Table 2. We presented two scenarios according to the availability (or not) of an HbA1 measured early in pregnancy, before the 14^th^ gestational week, and the real-life scenario, which included women with a first HbA1c measured at any time in pregnancy. The first model (Model 1, Table 2) included all women; the second and third models included only women with pregestational diabetes. Model 3 included all variables of model 2 plus the use of sulfonylurea. In this scenario, preeclampsia and HbA1c were associated with an increased risk of congenital anomaly (~ 80% and ~ 20%, respectively). The second scenario, a desirable one, included women with overt and pregestational diabetes with an HbA1c measured up to the 14^th^ gestational week (Model 4). Similar analyses were run and when only women with pregestational diabetes were analyzed, HbA1c was the only significant risk factor, imposing a 20% increased risk of any congenital anomaly. Sulfonylurea use (*p* = 0.078 in the univariable analyses) was not associated with congenital anomalies according to the multivariable models of women with pregestational diabetes, exclusively (Table 2, Model 3).

The potential interaction between obesity and diabetes, as risk factors for congenital anomalies, was tested: 10.6% of women with obesity and 12.0% in women without obesity in the group with overt diabetes (*p* = 0.998) and 14.3% compared to 17.9% in women in the pregestational diabetes group (*p* = 0.530) had a congenital anomaly.

**Table 2:** Risk factors for congenital anomalies in neonates of women with overt or pregestational type 2 diabetes according to two scenarios Porto Alegre, Brazil, 2005–2021

Scenario	REAL-LIFE						IDEAL		
First HbA1c	at any time in pregnancy						up to the 14^th^ gestational week		
	All women		Women with pregestational diabetes				All women		
	Model 1		Model 2		Model 3		Model 4		
	*n* = 538		*n* = 355		*n* = 355		*n* = 229		
Risk factor	aRR (95% CI)	p	aRR (95% CI)	p	aRR (95% CI)	p	aRR (95% CI)	p	
Pregestational diabetes	1.23 (0.76–1.99)	0.392					2.19 (0.71–6.81)	0.175	
Diabetes ≥ 6 years			1.17 (0.70–1.97)	0.545	1.16 (0.70–1.93)	0.568			
Obesity	0.82 (0.52–1.28)	0.382	0.87 (0.51–1.48)	0.604	0.90 (0.52–1.54)	0.695	1.17 (0.56–2.44)	0.676	
Preeclampsia	1.87 (1.23–2.85)	0.003	1.75 (1.08–2.82)	0.022	1.71 (1.06–2.76)	0.028	1.26 (0.66–2.40)	0.487	
HbA1c	1.21 (1.10–1.33)	< 0.001	1.22 (1.11–1.35)	< 0.001	1.20 (1.10–1.33)	< 0.001	1.22 (1.10–1.35)	< 0.001	
Sulfonylurea use					1.35 (0.81–2.25)	0.246			

aRR: adjusted relative risk; CI: confidence interval; HbA1c: glycated hemoglobin

Relative risks were calculated with multiple Poisson regression with robust estimates

Model 1, real life: adjusted for pregestational x overt diabetes, obesity (yes/no), pre-eclampsia (yes/no), HbA1c measured at any time in pregnancy (continuous variable)

Model 2, real life: included only women with pregestational diabetes and were adjusted for the variables in the Table (diabetes duration dichotomized as < 6 years and ≥ 6 years) and the other variables as above. Model 3 included all variables plus the information about the use of sulfonylurea (yes/no)

Model 4, ideal life: analyses as above, but performed only in the group with an HbA1c measured up to the 14^th^ gestational week as a continuous variable

No congenital anomaly was the reference category for the dependent variable

## Discussion

In this large cohort of women with known type 2 diabetes diagnosed before pregnancy and with overt diabetes, we found a high rate of congenital anomalies (13.8%), where most (93.6%) were major ones. The presence of congenital anomalies was associated with a high HbA1c measured in the first 14 weeks of gestation, and also at any time in pregnancy, according to the adjusted models.

There were several studies evaluating congenital anomalies in women with type 2 diabetes and their potential risk factors in recent decades. The frequencies of congenital anomalies varied across the time and population studied, with some of the figures accompanying the increased prevalence of type 2 diabetes. An official report from the Ministry of Health cites a low prevalence of congenital anomalies, 0.83%, in the general population [20], possibly an underestimation due to sub-notification, compared to rates of up to 6% worldwide [5]. In contrast, we found a high frequency in these high-risk participants. In six Brazilian studies including 238 pregnancies in women with type 2 diabetes, the rate of congenital anomalies was 8.8% [21–26]. Here, rates were almost fifteen times those described for the background Brazilian population and are at least twice the rate worldwide [5]. Findings of a multiethnic cohort of pregnant women with various degrees of hyperglycemia revealed low rates of congenital malformation in women with overt diabetes (1.1%) and those with early diagnosed gestational diabetes (1.4%), and a slightly greater incidence of congenital anomaly in women with a pre-pregnancy diagnosis of type 2 diabetes (2.1%) [27]. Low rates were also reported in the MOMPOD trial (2.8%) [28], in the United Kingdom (4.0%) [29], and in the MiTy trial (4.4%) [30]. The highest rate, 16.9%, was observed in an American cohort in the early 1990s [31].

In general, authors of the more recent studies have reported lower rates of congenital anomalies. However, our results were more in line with the studies conducted in the late 1990s [31, 32], even though our cohort was from the current century. The differences could be related to the participants’ profiles in other studies, as most of them took place in high-income countries, where preparedness for pregnancy, such as folate intake and adequate glycemic control, was more frequent, ~ 20.0% [29, 30, 33] when compared to the low-income scenario here, where preparedness was almost zero. Towner and colleagues reported several congenital anomalies in participants without preconception care counseling [31], a condition resembling ours.

Concerning the types of congenital anomalies, we found a similar pattern to that already reported: cardiac defects were the most frequent [29, 31]. Conversely, in the general picture of Brazil, limb defects outweighed cardiac and neurologic defects [20].

The high rates of congenital anomalies we found are probably multifactorial. First, we described an obesity rate of 77%. The mechanisms by which obesity is implicated as a factor for congenital anomalies are not fully understood. Obesity, similar to glycemia, apparently has a gradient-response effect on the development of congenital anomalies.

An unexpected association of preeclampsia to an increased risk of fetal malformation in women with a first HbA1c at any time in pregnancy, the real-life scenario in low-medium income countries (LMIC), was found, although not consistent when tested in the group with an early pregnancy first HbA1c. Current knowledge relates preeclampsia to a myriad of putative etiologies, with placental dysfunction and angiogenic imbalance as the most credited, which probably take place soon after embryo implantation or even before [15], and about the same time range of the hyperglycemia impact on the developing fetus. Diabetes is an acknowledged risk factor for preeclampsia, and an already unknown link between the two most important obstetric syndromes could exist.

The most important trigger is hyperglycemia, which is generally assessed by the measurement of preconception or first-trimester glycated hemoglobin (HbA1c), a valuable tool described for pregnant women with type 1 diabetes almost fifty years ago [34]. Higher first-trimester glycated hemoglobin was the only consistent and significant factor in our cohort, imposing a 23% increase in risk. There are many gaps in the understanding of mechanisms involving hyperglycemia. Hyperglycemia promotes increased glucose uptake via the glucose transporter GLUT2, leading to increased oxidative stress and cellular apoptosis through different pathways [7]. Hyperglycemia can also alter the expression of several genes involved in organogenesis. Most studies have reported values of HbA1c between 6.4% and 8.3% [22, 27, 29, 33], but in an American cohort, women delivering babies with major congenital anomalies had a mean HbA1c of 9.5% [31]. Only 23.7% of women in our cohort achieved pregnancy with an HbA1c < 6.5%, compared to 36.5% in another recent study [29]. Moreover, the diagnosis of overt diabetes adds to the risk of conceiving with inadequate metabolic control. Overt diabetes is not uncommon in pregnancy; 54% of women with type 2 diabetes included in one study were diagnosed during pregnancy [27], and rates of 13.5% and 21.4% were described in two randomized trials [28, 30]. In two other studies from the 1980s, the frequencies were similar to ours (~ 30%) [32, 35].

The synergistic or additive effects of obesity and hyperglycemia on the developing embryo could partially explain the high number of congenital anomalies, particularly cardiac anomalies [36] However, we could not detect an addictive effect of obesity and diabetes on congenital anomalies. Several other environmental factors have been implicated in the genesis of congenital anomalies, such as drugs, smoking, alcohol use, socioeconomic status, consumption of some foods, and pollutants, some of which are related to specific anomalies [6]. Here, few women reported smoking or using alcohol or other drugs. Few participants were screened for the use of alcohol or illicit substances since routine screening at childbirth admission was instituted in 2021 in only one of the two hospitals. None of these factors were associated with congenital anomalies, even in models adjusted for maternal age, obesity, schooling status (as a surrogate for socioeconomic status), and smoking status (data not shown). We did not collect information about food intake, but Brazilian women of childbearing age have a low frequency of fruit and vegetable consumption on five or more days per week [2]. We did not have information regarding environmental pollutant exposure in our sample.

Preconception care is crucial for reducing adverse outcomes in pregnancies complicated by hyperglycemia [8] and is associated with a decrease of 1.27% in the first trimester HbA1c [8]. Low rates of preparedness for pregnancy (15.6%), defined by folic acid supplementation, an HbA1c < 6.5%, and prevention of teratogenic medication use, were described in Irish women with type 2 diabetes [33]. We found low preparedness for pregnancy (less than 2.0%), as evidenced by rates of folic acid use (< 20.0%), late booking (most women already in the second trimester of pregnancy) and mainly, a high rate of overt diabetes (~ 30.0%), exposing the embryo to unknown hyperglycemia during organogenesis. The situation we had here is not unique. A low frequency of preconception care, evaluated by preconception or early pregnancy folate use, has also been reported in high-income countries [29] and was less than 40% according to a meta-analysis [8]. Regarding gestational age at booking, early arrival in the first trimester is reported in developed countries [29], whereas arrival at ~ 20 weeks of gestation is frequent in low-middle income countries (LMIC) [21, 23].

Our study has several merits. We evaluated a large cohort of participants with clinical characteristics of type 2 diabetes in a multiethnic population receiving prenatal care in the two major public regional hospitals over almost two decades. We identified a high percentage of participants with a first diagnosis of hyperglycemia during pregnancy, and we traced the characteristics of these participants, which were similar to those with known type 2 diabetes. Our cohort is likely representative of women of childbearing age with type 2 diabetes from LMIC, with a high prevalence of obesity and vast gaps in education. The high frequency of congenital anomalies, around ten times the rate of the background population, allowed a comprehensive appraisal of the associated risk factors.

Among the limitations, we can first cite the retrospective nature of the data collection. The results on medication use might be affected by incomplete information; we categorized all the missing information as “negative”. These could have led to an underestimation of several figures, mainly in data on folate use and potentially teratogenic medications, impacting the estimates of pregnancy preparedness. This decision could also have influenced analyses regarding tobacco, alcohol, and the use of illicit drugs. Conversely, not imputing negative values to missing variables could have led to results in the other direction by overestimating the frequencies: if we only used the yes/no answers for folic acid intake (yes *n* = 100, no *n* = 84, total *n* = 184), more than 50% of the sample would have taken the medicine, an unreal result. Our conviction that recording bias might have occurred due to the nature of both hospitals (public ones, with many registries performed by students or residents) led us to impute as “negative” all variables with missing values. For example, not infrequently, data about smoking and alcohol use were obtained from the nurses’ records. One solution to this limitation could be the implementation of institutional structured questionnaires into the electronic medical records to be filled in at the first prenatal appointment. Second, we had no access to genetic studies to evaluate cases of Maturity Onset Diabetes of the Young (MODY), which, however, accounts for very few cases of type 2 diabetes in pregnancy. Third, we did not confirm postpartum glycemic status in women with a diagnosis of overt diabetes, nor could we confirm the persistence of congenital anomalies, especially minor cardiac anomalies that might be transitory, nor those appearing later in childhood. Finally, we could not estimate the comparable risks of diabetes and obesity modifying the frequency of congenital anomalies due to the lack of a control group of women without obesity and diabetes. Nevertheless, we found no significant differences in the outcome when stratified simultaneously by obesity and type of diabetes.

## Conclusion

We found a high frequency of congenital anomalies in pregnant women with type 2 diabetes, which was primarily associated with poor glycemic control, revealing an almost universal lack of preconception care. Proxy indicators of deficient preconception care – high frequencies of obesity and undiagnosed diabetes, low frequency of folate supplementation, and late arrival at the high-risk prenatal care - were evident. An urgent call to action is mandatory for the reversal of this gray scenario.

### Electronic supplementary material

Below is the link to the electronic supplementary material.

Supplementary Material 1

## Data Availability

The datasets used and/or analyzed during the current study are available from the corresponding author upon reasonable request. The inquiries can be directed to the corresponding author (areichelt@hcpa.edu.br) or to Vânia Hirakata (vhirakata@hcpa.edu.br).

## Abbreviations

BMI: Body mass index
CAAE: Certificado de Apresentação de Apreciação Ética
CI: Confidence interval
EUROCAT: European Concerted Action on Congenital Anomalies and Twins
GLUT2: Glucose transporter 2
HbA1c: Glycated hemoglobin
HCPA: Hospital de Clínicas de Porto Alegre
HNSC: Hospital Nossa Senhora da Conceição
LMIC: Low-medium income countries
MiTY: Metformin in Women with Type 2 Diabetes in Pregnancy Trial
MODY: Maturity-Onset Diabetes of Young
MOMPOD: Medical Optimization of Management of Type 2 Diabetes Complicating Pregnancy
n: Number
NICU: Neonatal intensive care unit
PSS_Health: Power and Sample Size for Health Researchers
RR: Relative risk
SD: Standard deviation
STROBE: Strengthening the Reporting of Observational Studies in Epidemiology
