# Supplementary material for: Congenital anomalies in pregnancies with overt and pregestational type 2 diabetes: a gray portrayal from a cohort in Brazil
Source: Diabetol Metab Syndr. 2024 Jul 11;16:157. doi: 10.1186/s13098-024-01376-y (PMC11238503; doi:10.1186/s13098-024-01376-y)
Supplement: Supplementary file 1 — Supplementary Material 1 [file 13098_2024_1376_MOESM1_ESM.docx]

Additional File 1

| Suppl Table 1. Congenital anomalies and associated factors in pregnancies complicated by overt and pregestational type 2 diabetes, by diagnosis classification  Porto Alegre, Brazil, 2005-2021 | | | | |
| --- | --- | --- | --- | --- |
| Characteristic | Diabetes | | | p |
|  | All | Overt | Pregestational |  |
|  | n=567 (100.0) | n=191 (33.7) | n=376 (66.3) |  |
| Center |  |  |  | 0.644 |
| HCPA | 258 (45.5) | 90 (47.1) | 168 (44.7) |  |
| HNSC | 309 (54.5) | 101 (52.9) | 208 (55.3) |  |
| Age (years) | 32.8 ± 5.9 | 31.9 ± 6.1 | 33.2 ± 5.7 | 0.007 |
| White skin color (yes) | 391 (69.0) | 132 (69.1) | 269 (68.9) | >0.999 |
| Schooling (≤ 11 years) (yes) | 538 (94.9) | 185 (96.9) | 353 (93.9) | 0.187 |
| Smoking (yes) | 50 (8.8) | 25 (13.1) | 25 (6.6) | 0.016 |
| Diabetes complications (yes) | 36 (6.3) | 2 (1.0) | 34 (9.0) | <0.001 |
| Chronic hypertension (yes) | 147 (25.9) | 30 (15.7) | 117 (31.1) | <0.001 |
| BMI categories |  |  |  | 0.557 |
| normal | 55 (10.0) | 19 (10.4) | 36 (9.9) |  |
| overweight | 107 (19.5) | 31 (16.9) | 76 (20.8) |  |
| obesity | 386 (70.4) | 133 (72.7) | 253 (69.3) |  |
|  | 548 | 183 | 365 |  |
| Folate use (yes) | 100 (17.6) | 26 (13.6) | 74 (19.7) | 0.094 |
| Treatment at conception |  |  |  | <0.001 |
| none | 228 (40.6) | 191 (100.0) ^a^ | 37 (10.0) ^b^ |  |
| diet | 21 (3.7) | 0 ^a^ | 21 (5.7)^b^ |  |
| oral antidiabetics | 240 (42.7) | 0 ^a^ | 240 (64.7)^b^ |  |
| insulin | 27 (4.8) | 0^a^ | 27 (7.3)^b^ |  |
| insulin + oral antidiabetics | 46 (8.2) | 0^a^ | 46 (12.4)^b^ |  |
|  | 562 | 191 | 371 |  |
| Mean HbA1c before 14 weeks | 7.8 (1.8) | 7.4 (1.4) | 7.9 (1.9) | p=0.027 |
| no anomaly* | 7.6 (1.6) | 7.4 (1.3) | 7.7 (1.7) |  |
|  | 204 | 46 | 158 |  |
| major anomaly* | 9.0 (2.4) | 8.5 (1.6) | 9.0 (2.5) |  |
|  | 33 | 2 | 31 |  |
| minor anomaly* | 7.3 (2.2) | 5.4 | 8.3 (2.1) |  |
|  | 3 | 1 | 2 |  |
|  | 240 | 49 | 191 |  |
| HbA1c < 6.5% before 14 weeks | 57 (23.8) | 11 (22.4) | 46 (24.1) | 0.959 |
|  | 240 | 49 | 191 |  |
| Type of anomaly |  |  |  | 0.014 |
| no anomaly | 489 (86.2) | 171 (89.5) ^a^ | 318 (84.6) ^a^ |  |
| cardiac | 43 (7.6) | 7 (3.7) ^a^ | 36 (9.6) ^b^ |  |
| renal/urologic | 8 (1.4) | 5 (2.6) ^a^ | 3 (0.8) ^a^ |  |
| neurologic | 9 (1.6) | 0 (0.0) ^a^ | 9 (2.4) ^b^ |  |
| musculoskeletal | 5 (0.9) | 3 (1.6) ^a^ | 2 (0.5) ^a^ |  |
| cleft palate | 1 (0.2) | 0 (0.0) ^a^ | 1 (0.3) ^a^ |  |
| multiple | 12 (2.1) | 5 (2.6) ^a^ | 7 (1.9) ^a^ |  |
|  | 566 | 191 | 375 |  |

HCPA: Hospital de Clínicas de Porto Alegre; HNSC: Hospital Nossa Senhora da Conceição; HbA1c: glycated hemoglobin; BMI: body mass index

ICD for the anomalies (all major anomalies; minor anomalies are indicated):

cardiac: Q25.1 (n=1), Q24.1 (n=1), Q23.0 (n=3, minor), Q 22.5 (n=1), Q21.3 (n=1), Q21.1 (n=9), Q21.0 (n=23), Q20.8 (n=1), Q20.3 (n=1), Q20.0 (n=2);

renal/urologic: Q63.8 (n=2), Q 62.0 (n=1), Q61.9 (n=2), Q64.8 (n=1), Q 60 (n=1), Q53.0 (n=1, minor);

neurologic: Q 79.5 (n=1), Q 76.4 (n=2), Q5.9 (n=1), Q6.8 (n=1), Q4.2 (n=2), Q4.0 (n=1), Q0.0 (n=1);

musculoskeletal: Q69.0 (n=2), Q66.6 (n=1), Q17.0 (n=1, minor), Q79.2 (n=1);

cleft palate Q35.0 (n=1);

multiple Q89.7 (n=12)

ICD: International Classification of Diseases (10^th^ version)

The data are presented as the mean (standard deviation) or n (%)

The total number of cases is in the caption of the Table. We added a line with the actual number of cases analyzed under the results of the variables with missing data.

p values were calculated with the χ^2^ test for categorical variables and the Student’s t-test for continuous variables.

^a, b^ Different letters in the same line indicate statistical differences analyzed by the Z-test for proportion comparisons, corrected by Bonferroni (p<0.05).

| Suppl Table 2. Number of congenital anomalies across time in babies of women with overt and pregestational diabetes.  Porto Alegre, Brazil, 2005-2021 | | | | |
| --- | --- | --- | --- | --- |
|  | All babies | | | |
| Year | Congenital anomaly (yes) | | Total number of babies | |
|  | n | % | n | % |
| 2005 | 2 | 28.6 | 7 | 100 |
| 2006 | 2 | 25.0 | 8 | 100 |
| 2007 | 0 | 0.0 | 8 | 100 |
| 2008 | 0 | 0 | 2 | 100 |
| 2009 | 0 | 0 | 12 | 100 |
| 2010 | 5 | 20.0 | 25 | 100 |
| 2011 | 5 | 15.6 | 32 | 100 |
| 2012 | 4 | 7.3 | 55 | 100 |
| 2013 | 6 | 12.8 | 47 | 100 |
| 2014 | 9 | 16.4 | 55 | 100 |
| 2015 | 2 | 4.3 | 46 | 100 |
| 2016 | 6 | 15.0 | 40 | 100 |
| 2017 | 7 | 14.3 | 49 | 100 |
| 2018 | 7 | 12.3 | 57 | 100 |
| 2019 | 7 | 10.9 | 64 | 100 |
| 2020 | 7 | 15.9 | 44 | 100 |
| 2021 | 9^b^ | 56.3 | 16 | 100 |
| Total | 78 | 13.8 | 567 | 100 |

p values calculated with the χ^2^ test

^b^ indicates statistical differences in the same line analyzed by the Z-test for proportion comparisons, corrected by Bonferroni (p<0.05) (2021, n=16 babies, 9 with congenital anomalies, 7 without, p=0.002).

| Suppl Table 3. Characteristics of women with pregestational type 2 diabetes by congenital anomalies  Porto Alegre, Brazil, 2005-2021 | | | | |
| --- | --- | --- | --- | --- |
| Characteristic |  | Congenital anomaly | | p |
|  | All  n= 376 (100.0) | Yes  n=58 (15.4) | No  n=318 (84.6) |  |
| Baseline characteristics | | | | |
| Center |  |  |  | 0.327 |
| HCPA | 168 (44.7) | 22 (13.1) | 146 (86.9) |  |
| HNSC | 208 (55.3) | 36 (17.3) | 172 (82.7) |  |
| Age (years) | 33.3 ± 5.7 | 32.8 ± 6.4 | 33.4 ± 5.6 | 0.465 |
| Duration of diabetes |  |  |  | 0.051 |
| up to 5 years | 251 (67.1) | 32 (12.7) | 219 (87.3) |  |
| ≥ 6 years | 123 (32.9) | 26 (21.1) | 97 (78.9) |  |
|  | 374 | 58 | 316* |  |
| Skin color |  |  |  | 0.889 |
| white | 259 (69.9) | 39 (15.1) | 220 (84.9) |  |
| non-white | 117 (31.1) | 19 (16.2) | 98 (83.8) |  |
| Schooling |  |  |  | 0.571 |
| ≤ 11 years | 353 (93.9) | 1. (15.0) | 300 (85.0) |  |
| > 11 years | 23 (6.1) | 5 (21.7) | 18 (78.3) |  |
| Smoking |  |  |  | 0.838 |
| yes | 25 (6.6) | 3 (12.0) | 22 (88.0) |  |
| no | 351 (93.4) | 55 (15.7) | 296 (84.3) |  |
| Pre-pregnancy treatment |  |  |  | 0.738 |
| none | 37 (10.0) | 6 (16.2) | 31 (83.8) |  |
| diet only | 21 (5.7) | 1 (4.8) | 20 (95.2) |  |
| oral medication | 240 (64.6) | 39 (16.3) | 201 (83.7) |  |
| insulin | 27 (7.3) | 4 (14.8) | 23 (85.2) |  |
| oral + insulin | 46 (12.4) | 7 (15.2) | 39 (84.8) |  |
|  | 371 | 57 | 314 |  |
| Folic acid intake |  |  |  | 0.439 |
| yes | 74 (19.7) | 14 (18.9) | 60 (81.1) |  |
| no | 56 (14.9) | 6 (10.7) | 50 (89.3) |  |
| no information | 246 (65.4) | 38 (15.4) | 208 (84.6) |  |
| Anti-hypertensive drugs |  |  |  | 0.200 |
| yes | 86 (22.9) | 9 (10.5) | 77 (89.5) |  |
| no | 290 (77.1) | 49 (16.9) | 241 (83.1) |  |
| Statins |  |  |  | >0.999 |
| yes | 14 (3.7) | 2 (14.3) | 12 (85.7) |  |
| no | 362 (96.3) | 56 (15.5) | 306 (84.5) |  |
| Metformin** |  |  |  | 0.903 |
| yes | 260 (69.1) | 41 (15.8) | 219 (84.2) |  |
| no | 116 (30.9) | 17 (14.7) | 99 (85.3) |  |
| Sulfonylurea** |  |  |  | 0.078 |
| yes | 92 (24.5) | 20 (21.7) | 72 (78.3) |  |
| no | 284 (75.5) | 38 (13.4) | 246 (86.6) |  |
| Diabetes complications |  |  |  | 0.532 |
| yes | 34 (9.0) | 7 (20.6) | 27 (79.4) |  |
| no | 342 (91.0) | 51 (14.9) | 291 (85.1) |  |
| Chronic hypertension |  |  |  | 0.866 |
| yes | 117 (31.1) | 17 (14.5) | 100 (85.5) |  |
| no | 259 (68.9) | 41 (15.8) | 218 (84.2) |  |
| BMI |  |  |  |  |
| Pregestational (kg/m^2^) | 34.1±7.6 | 33.7 ± 7.6 | 34.2±7.6 | 0.632 |
| categories |  |  |  | 0.530 |
| no obesity | 112 (30.7) | 20 (17.9) | 92 (82.1) |  |
| obesity | 253 (69.3) | 37 (14.6) | 216 (85.4) |  |
|  | 365 | 57 | 308 |  |
| Gestational age at booking (weeks) | 18.6 ± 7.7 | 20.2 ± 8.2 | 18.3 ± 7.6 | 0.091 |
| First HbA1c | 7.4 ± 1.7 | 8.3 ± 2.2 | 7.2 ± 1.5 | <0.001 |
|  | 373 | 58 | 315 |  |
| HbA1c before 14 weeks |  |  |  | 0.517 |
| < 6.5% | 46 (24.1) | 6 (13.0) | 40 (87.0) |  |
| ≥ 6.5% | 145 (75.9) | 27 (18.6) | 118 (81.4) |  |
|  | 191 | 33 | 158 |  |
| Pre-eclampsia |  |  |  | 0.035 |
| yes | 128 (34.6) | 26 (20.3) | 102 (79.7) |  |
| no | 242 (65.4) | 28 (11.6) | 214 (88.4) |  |
|  | 370 | 54 | 316 |  |

*two women with pregestational diabetes with unknown diabetes duration

**analyses performed only for women with known pregestational diabetes

HCPA: Hospital de Clínicas de Porto Alegre; HNSC: Hospital Nossa Senhora da Conceição; BMI: body mass index; HbA1c: glycated hemoglobin

The total number of cases is in the caption of the Table. We added a line with the actual number of cases analyzed under the results of the variables with missing data.

The data are presented as the mean (standard deviation) or n (%)

p values were calculated with the χ^2^ test for categorical variables and the Student’s t-test for continuous variables.

| Suppl Table 4. Characteristics of women with pregestational type 2 diabetes by HbA1c measured up to the 14^th^ gestational week  Porto Alegre, Brazil, 2005-2021 | | | | |
| --- | --- | --- | --- | --- |
| Characteristic |  | Congenital anomaly | | p |
|  | All  n=191 (100.0) | Yes  n=33 (17.3) | No  n=158 (82.7) |  |
| Baseline characteristics | | | | |
| Center |  |  |  | 0.838 |
| HCPA | 87 (45.5) | 14 (16.1) | 73 (83.9) |  |
| HNSC | 104 (55.5) | 19 (18.3) | 85 (81.7) |  |
| Age (years) | 33.5 ± 5.8 | 33.1 ± 7.0 | 33.6 ± 5.6 | 0.632 |
| Diabetes diagnosis | 190 | 33 | 157* | 0.051 |
| up to 5 years | 123 (64.7) | 16 (13.0) | 107 (87.0) |  |
| ≥ 6 years | 67 (35.3) | 17 (25.4) | 50 (74.6) |  |
| Skin color |  |  |  | 0.785 |
| white | 134 (70.2) | 22 (16.4) | 112 (83.6) |  |
| non-white | 57 (29.8) | 11 (19.3) | 46 (80.7) |  |
| Schooling |  |  |  | 0.705 |
| ≤ 11 years | 1. (91.1) | 29 (16.7) | 145 (83.3) |  |
| > 11 years | 17 (8.9) | 4 (23.5) | 13 (76.5) |  |
| Smoking |  |  |  | 0.651 |
| yes | 12 (6.3) | 1 (8.3) | 11 (91.7) |  |
| no | 179 (93.7) | 32 (17.9) | 147 (82.1) |  |
| Pre-pregnancy treatment |  |  |  | 0.607 |
| none | 21 (11.2) | 5 (23.8) | 16 (76.2) |  |
| diet only | 4 (2.1) | 0 (0.0) | 4 (100.0) |  |
| oral medication | 124 (65.9) | 21 (16.9) | 103 (83.1) |  |
| insulin | 12 (6.4) | 3 (25.0) | 9 (75.0) |  |
| oral + insulin | 27 (14.4) | 3 (11.1) | 24 (88.9) |  |
|  | 188 | 32 | 156 |  |
| Folic acid intake |  |  |  | 0.707 |
| yes | 55 (28.8) | 10 (18.2) | 45 (81.8) |  |
| no | 26 (13.6) | 3 (11.5) | 23 (88.5) |  |
| no information | 110 (57.6) | 20 (18.2) | 90 (81.8) |  |
| Anti-hypertensive drugs |  |  |  | 0.565 |
| yes | 45 (23.6) | 6 (13.3) | 39 (86.7) |  |
| no | 146 (76.4) | 27 (18.5) | 119 (80.5) |  |
| Statins |  |  |  | 0.845 |
| yes | 10 (5.2) | 1 (10.0) | 9 (90.0) |  |
| no | 181 (94.8) | 32 (17.7) | 149 (82.3) |  |
| Metformin |  |  |  | 0.490 |
| yes | 134 (70.2) | 21 (15.7) | 113 (84.3) |  |
| no | 57 (29.8) | 12 (21.1) | 45 (78.9) |  |
| Sulfonylurea |  |  |  | 0.111 |
| yes | 51 (26.7) | 13 (25.5) | 38 (74.5) |  |
| no | 140 (73.3) | 20 (14.3) | 120 (85.7) |  |
| Diabetes complications |  |  |  | 0.574 |
| yes | 26 (13.6) | 6 (23.1) | 20 (76.9) |  |
| no | 165 (86.4) | 27 (16.4) | 138 (83.6) |  |
| Chronic hypertension |  |  |  | 0.785 |
| yes | 57 (29.8) | 11 (19.3) | 46 (80.7) |  |
| no | 134 (70.2) | 22 (16.4) | 112 (83.6) |  |
| BMI |  |  |  |  |
| mean (kg/m^2^) | 33.9 ± 7.3 | 34.4 ± 7.7 | 33.8 ± 7.2 | 0.673 |
| categories |  |  |  | 0.975 |
| obesity | 129 (70.0) | 22 (17.1) | 107 (82.9) |  |
| no obesity | 58 (30.0) | 10 (17.2) | 48 (82.8) |  |
|  | 187 | 32 | 155 |  |
| Gestational age at booking (weeks) | 13.9 ± 5.7 | 16.2 ± 7.0 | 13.4 ± 5.0 | 0.033 |
| Mean HbA1c | 7.9 ± 1.9 | 9.0 ± 2.4 | 7.7 ± 1.7 | 0.007 |
| HbA1c before 14 weeks |  |  |  | 0.517 |
| < 6.5% | 46 (24.1) | 6 (13.0) | 40 (87.0) |  |
| ≥ 6.5% | 145 (75.9) | 27 (18.6) | 118 (81.4) |  |
| Pre-eclampsia |  |  |  | 0.626 |
| yes | 66 (35.7) | 12 (18.2) | 54 (81.8) |  |
| no | 119 (64.3) | 17 (14.3) | 102 (85.7) |  |
|  | 185 | 29 | 156 |  |

* one women with pregestational diabetes with unknown timing of diagnosis

HbA1c: glycated hemoglobin; HCPA: Hospital de Clínicas de Porto Alegre; HNSC: Hospital Nossa Senhora da Conceição; BMI: body mass index

The total number of cases is in the caption of the Table. We added a line with the actual number of cases analyzed under the results of the variables with missing data.

The data are presented as the mean (standard deviation) or n (%)

p values were calculated with the χ^2^ test for categorical variables and the Student’s t-test for continuous variables.

^a, b^ Different letters in the same line indicate statistical differences analyzed by the Z-test for proportion comparisons, corrected by Bonferroni (p<0.05).


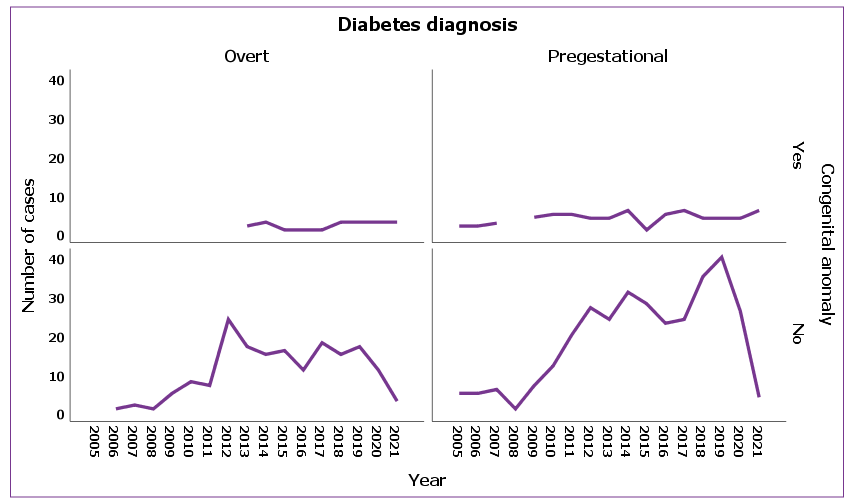


Suppl Figure 1. Number of cases of congenital anomalies according to the year of the first prenatal appointment by the maternal type of diabetes

Porto Alegre, Brazil, 2005-2021
